# Septic Shock, Adult Respiratory Distress Syndrome, and Disseminated Intravascular Coagulopathy Following Midtrimester Genetic Amniocentesis

**DOI:** 10.1155/S1064744997000707

**Published:** 1997

**Authors:** J. R. Johnson, P. G. Stubblefield, M. A. Hamid, J. Kasznica

**Affiliations:** 1 Boston University School of Medicine and Boston Medical Center Boston MA USA; 2 One Boston Medical Center Place Maternity 3 Boston MA 02118 USA

## Abstract

*Background:* Midtrimester genetic amniocentesis is a commonly performed procedure, with acknowledgment of some risk to mother and fetus.

*Case:* We present an unusual case of midtrimester genetic amniocentesis with bowel injury and resulting septic shock, adult respiratory distress syndrome, and disseminated intravascular coagulation. A total abdominal hysterectomy and bilateral salpingoophorectomy were required for resolution of sepsis. The patient also required prolonged ventilatory support postoperatively.

*Conclusion:* Although relatively safe, genetic amniocentesis can result in serious morbidity, and attention to technique should be maintained.


Infectious Diseases in Obstetrics and Gynecology 5:386–390 (1997)
© 1998 Wiley-Liss, Inc.

### Septic Shock, Adult Respiratory Distress Syndrome, and Disseminated Intravascular Coagulopathy Following Midtrimester Genetic Amniocentesis

J.R. Johnson,* P.G. Stubblefield, M.A. Hamid, and J. Kasznica
*Boston University School of Medicine and Boston Medical Center, Boston, MA*

ABSTRACT

***Background:*** **Midtrimester genetic amniocentesis is a commonly performed procedure, with acknowledgment of some risk to mother and fetus.**

***Case:*** **We present an unusual case of midtrimester genetic amniocentesis with bowel injury and resulting septic shock, adult respiratory distress syndrome, and disseminated intravascular coagulation. A total abdominal hysterectomy and bilateral salpingoophorectomy were required for resolution of sepsis. The patient also required prolonged ventilatory support postoperatively.**

***Conclusion:*** **Although relatively safe, genetic amniocentesis can result in serious morbidity, and attention to technique should be maintained. Infect. Dis. Obstet. Gynecol. 5:386–390, 1997.**
© 1998 Wiley-Liss, Inc.

KEY WORDS
genetic screening, bowel injury, adult respiratory distress syndrome

Genetic amniocentesis for a variety of indications has become routine practice in our country. Written informed consent is usually obtained prior to the procedure and typically includes an acknowledgment that there is some small degree of risk to the fetus. That there can also be life-threatening risk to the woman is not a part of our usual consent process. We describe an unusual case of severe sepsis with long-term morbidity following genetic amniocentesis.

### CASE REPORT

A 34-year-old woman, gravida six, para 2032, at approximately 20 weeks gestation arrived at a suburban hospital reporting fever, chills, and abdominal pain. The patient reported no fetal movement in the preceding 24 hours. She had undergone an amniocentesis in her obstetrician's office two days prior to admission because of a family history of Down syndrome. The amniocentesis had required two attempts under continuous ultrasound guidance. The amniotic fluid was noted to be clear at the time of the procedure. Over the two days subsequent to the amniocentesis, she developed malaise, fever, and chills. When examined at the hospital, the patient had a temperature of 39.7°C and a heart rate of 147 bpm. Her lower abdomen was tender. There were two small marks beneath the umbilicus, overlying the uterine fundus, consistent with the site of entry of the amniocentesis needle. Ultrasound examination confirmed intrauterine demise of a fetus at 20 weeks of gestation. The patient soon became acutely anxious and short of breath, and her $O_2$ saturation (percent oxygen saturation) dropped into the low 80s on room air.

The patient's blood pressure was 90/51 mmHg, and her heart rate was in the 160-bpm range. While the patient was breathing 100% oxygen by face mask, her arterial blood gas measurement was pH 7.36, her $pCO_2$ was 20 mmHg, her $pO_2$ was 86 mmHg, and her $O_2$ saturation was 97%. Rales and ronchi were apparent over both lung fields. She

*Correspondence to: Dr. Jeffrey R. Johnson, One Boston Medical Center Place, Maternity 3, Boston, MA 02118.
Received 30 October 1997
Accepted 12 February 1998

was moved to the surgical intensive care unit, where she was immediately intubated for acute respiratory distress. Pink frothy secretions were noted in the endotracheal tube.

Antibiotic therapy was begun with intravenous ampicillin (2 g every four hours), clindamycin (900 mg every eight hours), and gentamicin (a 140-mg loading dose followed by a maintenance dose of 100 mg every eight hours). A neosynephrine drip was started to maintain the patient's falling blood pressure. Neosynephrine was chosen as the pressor agent over dopamine because it has exclusive alpha vasopressor properties with no increase in heart rate. The patient had a heart rate in the 160-bpm range, and cardiac output may have been compromised if the heart rate increased any further. Her hematocrit fell from 34% on admission to 17.9% two hours later, and her platelet count fell from 109,000 to 61,000. Coagulation studies were performed when she arrived at the surgical intensive care unit, and the international normalization ratio was 2.41 with an activated partial thromboplastin time of 59.2 seconds. Disseminated intravascular coagulopathy (DIC) secondary to sepsis was diagnosed. The patient received a transfusion of five units of packed red blood cells and four units of fresh frozen plasma. Increasing doses of neosynephrine were required to maintain blood pressure, and dobutamine was added at 6 μg per minute for additional pressor support. A Swan-Ganz catheter and arterial line were placed for intravascular monitoring. A chest X-ray showed acute pulmonary edema, and no infiltrates were seen. Induction of labor was begun with intravenous oxytocin, 50 units in 1,000 ml of lactated Ringer's solution, initially infused at 100 ml per hour. Because she began developing acidosis in spite of maximal respiratory support, the patient was transferred by ambulance to a tertiary facility, accompanied by a physician.

Upon arrival at the tertiary facility, it was determined that the patient had blood gases of pH 7.33, a $pCO_2$ of 73 mmHg, a $pO_2$ of 74 mmHg, and percent $O_2$ saturation of 91.7% while receiving 100% oxygen. Her blood pressure was 90/40 mmHg, and her pulse was in the 140-bpm-range. Chest X-ray confirmed either acute pulmonary edema or early adult respiratory distress syndrome (ARDS). Severe sepsis with shock, respiratory failure, and DIC were attributed to chorioamnionitis secondary to amniocentesis with presumed passage of the needle through the bowel. A positive end expiratory pressure of 15 mmHg was required to maintain oxygen saturation above 90%, but a persistent acidosis developed with a pH of 7.24 to 7.26. The oxytocin regimen was changed to the high-dose oxytocin protocol described by Winkler, et al.:[1] 50 units of oxytocin in 500 ml of normal saline given over three hours, then, after one hour off oxytocin, resumed with 100 units of oxytocin in 500 ml of solution given over three hours. Five medium *laminaria japonicum* tents were placed in the cervical canal. At 10 hours after arrival at the tertiary center, the patient was taken to the operating room where a dilatation and evacuation procedure was accomplished under general anesthesia. The cervix was five cm dilated when the laminaria were removed, and the 336-g fetus and placenta were extracted essentially intact. The uterine contents had a pronounced fecal smell. Uterine cultures were obtained.

Over the next several hours, the patient's condition improved somewhat, and the systemic acidosis improved, with the blood pH rising to 7.41 to 7.43. A positive end expiratory pressure of 15 mmHg and $FiO_2$ of 70–80% were needed, and the patient remained febrile at 38.8°C. Her heart rate remained 130–150 bpm, and infusions of dopamine, neosynephrine, and dobutamine were needed to maintain the blood pressure at 75–110/40–70 mmHg. Cardiac output was low at 5.5 to 7 l/min, and pulmonary capillary wedge pressure was 17–20 mmHg. Echocardiography revealed hypokinetic ventricles. The hematocrit fell to 14%, and the white blood cell count was 24,300/$mm^3$. Because of the continuing severe sepsis and lack of improvement, laparotomy was performed under general anesthesia at 24 hours after uterine evacuation. A midline incision was made, and abundant dark yellow ascitic fluid but no free pus was found upon entry into the peritoneal cavity. The uterus was well-contracted at the size of 16 weeks gestation, with an 8 × 10-cm soft ecchymosis present on the uterine fundus. Examination of the entire small and large intestine was performed. A small area of purulent exudate was discovered on the sigmoid colon where it overlay the uterine fundus. Beneath this exudate was an erythematous area on the bowel serosa 3 × 2 mm in diameter, consistent with a needle puncture. There was no similar wound of

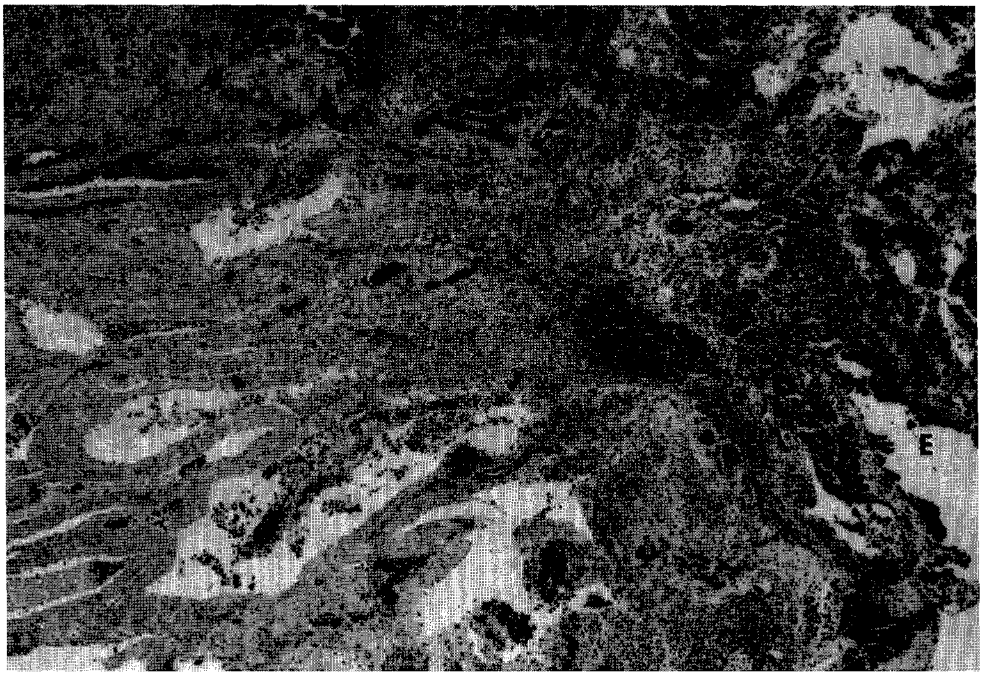

Fig. 1. Low-power view showing amniocentesis needle tract area, which was contiguous from the uterine serosa to the endometrial cavity. Note the extensive hemorrhagic necrosis and inflammation of the uterine tissue from the endometrial aspect (E). (H.E. × 20, high contrast photo micrograph.)

exit on the opposite side. Air was injected via a rectal catheter into the sigmoid colon and no air leak from the puncture site was observed while the sigmoid colon was held in the saline-filled pelvis. The puncture site was inverted using 3.0 silk sutures through the seromuscular layers in an interrupted fashion. Total abdominal hysterectomy was performed without complication. Bilateral salpingoophorectomy was also performed, as there was evidence at the time of laparotomy that the ovarian veins contained thrombi and purulent material and may have been an additional source of sepsis. A large Jackson-Pratt drain was brought out through a separate stab wound and the rectus and fascia closed in a single layer. The skin and subcutaneous layers were left open, with preplaced mattress sutures left untied for a delayed primary closure, and the wound was packed with sterile gauze. Gross pathologic exam of the enlarged 480-g uterus revealed a purulent tract with adjacent abscess formation. The tract extended from the uterine surface to the endometrial cavity. Hemorrhagic necrosis, septic thrombi of the venous channels, and abscess formation were confirmed by microscopic examination (see Figures 1, 2).

The posthysterectomy course was marked by steady resolution of hypotension and improvement in cardiac status. Blood cultures taken on admission at the first hospital grew *E. coli*, and blood cultures obtained on admission from the second hospital grew *E. coli* and Enterobacter. Cultures from the uterine cavity obtained at the initial uterine evacuation revealed *Enterobacter cloacae*, Gram-negative bacilli, Clostridium species, and two Bacteroides species. Broad spectrum antibiotics were continued through the patient's 17th day in the hospital. The skin was closed by tying the preplaced sutures on the patient's sixth day in the hospital.

A tracheostomy was placed 22 days postoperatively after an unsuccessful attempt to wean the patient from the ventilator. The patient was taken off ventilator support after 32 days but required supplemental oxygen. The wound healed without incident. Intensive care was discontinued after 34 days, and the patient was discharged to a rehabilitation facility after 37 days for physical and respiratory therapy. On follow-up visit six weeks after discharge, the patient appeared well. She had resumed her normal daily activities without dyspnea. There was no short-term or long-term memory loss,

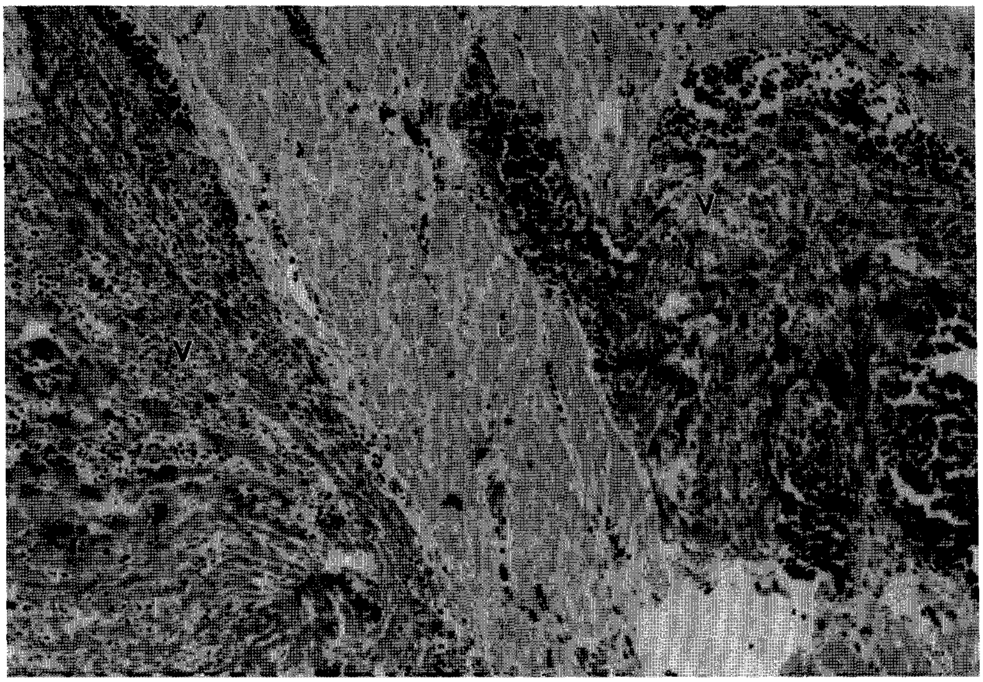

Fig. 2. Higher-power view showing the relationship of thrombosed venous channels (V) to the necrotic smooth muscle of the myometrium. (H.E. × 100, high contrast photomicrograph.)

except for the immediate events surrounding her admission to the intensive care unit and surgery. Physical examination revealed a normal posthysterectomy state.

### DISCUSSION

The findings at laparotomy, the patient's hospital course, and the results of the uterine cultures are most consistent with inoculation of the uterine cavity with bowel organisms during the attempted amniocentesis. Obstetric texts list amnionitis as a possible complication of amniocentesis, stating that this occurs in less than 0.1% of amniocentesis[2] or that symptomatic amnionitis occurs "rarely" after second-trimester amniocentesis,[3, 4] which may result in fetal demise and septic abortion. However, cases in which severe sepsis with ARDS caused by second-trimester amniocentesis seem inadequately reflected in standard obstetrical texts. We were able to find only one previous case report of uterine infection caused by *Clostridium perfringens* following amniocentesis.[5] That case was also complicated by ARDS and acute renal failure due to overwhelming sepsis. The patient did not require hysterectomy and survived after a similarly long illness. The authors hypothesized that the amniocentesis had caused a high rupture of the fetal membranes with ascending infection of Clostridium from the vagina but admit the possibility that the amniocentesis needle could have traversed the colon, causing fecal contamination of the uterine cavity. *C. perfringens* has been found in vaginal and cervical cultures in 4–10% of healthy women.[5]

The mortality associated with ARDS is 50% and is higher in cases of severe sepsis and DIC.[6] Clostridial infection with bacteremia is associated with a mortality of 70%.[6] In our case, we proceeded to uterine evacuation after 10 hours of preparation with high-dose oxytocin and laminaria, following accepted principles of management of intrauterine sepsis.[7] In retrospect, this treatment was inadequate, as there was an abscess in the uterine wall. The myometrium was therefore a continuous source of infection. As noted by Faro and Pearlman,[8] antibiotic therapy for septic abortion does not suffice if there is myometrial abscess or necrotizing myometritis. Immediate hysterectomy after initial stabilization might have led to more rapid recovery and less long-term morbidity. Infection is one of the possible complications of amniocentesis, but fortunately sepsis of the magnitude reported here is clearly uncommon. The case presented here signals a need for continued attention to details of technique.

### REFERENCES

1. Winkler CL, Gray S, Hauth J: Mid-trimester labor induction: Concentrated oxytocin compared with prostaglandin E2 suppositories. Obstet Gynecol 77:297–300, 1991.
2. Simpson JL: Genetic counseling and prenatal diagnosis. In Gabbe S, Niebyl J, Simpson J (eds): Obstetrics: Normal and Problem Pregnancies. 2nd ed. New York: Churchill Livingstone, pp 269–298, 1991.
3. Simpson JL, Elias S: Prenatal diagnosis of genetic disorders. In Creasy R, Resnik R (eds): Maternal-Fetal Medicine: Principles and Practice. 3rd ed. Philadelphia: W. B. Saunders Company, pp 61–88, 1994.
4. Prenatal diagnosis and invasive techniques to monitor the fetus. In Cunningham FG, MacDonald PC, Leveno K, Gant NF, Gilstrap LC III (eds): Williams Obstetrics. 19th ed. Norwalk, Connecticut: Appleton & Lange, pp 939–957, 1993.
5. Hovav Y, Hornstein E: Sepsis due to Clostridium prefringens after second-trimester amniocentesis [letter]. Clin Infect Dis 21:235–236, 1995.
6. Lung injury and pulmonary edema. In Marino P (ed): The ICU Book. Philadelphia: Lea & Febiger, pp 293–308, 1991.
7. Stubblefield P, Grimes D. Septic abortion. N Engl J Med 35:310–314, 1994.
8. Infections and abortion. In Faro S, Pearlman M (eds): Current Topics in Obstetrics and Gynecology. New York: Elsevier, p. 86, 1992.
